# Emerging Evidence for Cannabis' Role in Opioid Use Disorder

**DOI:** 10.1089/can.2018.0022

**Published:** 2018-09-01

**Authors:** Beth Wiese, Adrianne R. Wilson-Poe

**Affiliations:** ^1^ Department of Psychology, University of Missouri–St. Louis, St. Louis, Missouri.; ^2^ Department of Anesthesiology, Pain Center, Washington University School of Medicine, St. Louis, Missouri.

**Keywords:** cannabis, opioid addiction, opioid treatment, relapse prevention

## Abstract

**Introduction: **The opioid epidemic has become an immense problem in North America, and despite decades of research on the most effective means to treat opioid use disorder (OUD), overdose deaths are at an all-time high, and relapse remains pervasive.

**Discussion:** Although there are a number of FDA-approved opioid replacement therapies and maintenance medications to help ease the severity of opioid withdrawal symptoms and aid in relapse prevention, these medications are not risk free nor are they successful for all patients. Furthermore, there are legal and logistical bottlenecks to obtaining traditional opioid replacement therapies such as methadone or buprenorphine, and the demand for these services far outweighs the supply and access. To fill the gap between efficacious OUD treatments and the widespread prevalence of misuse, relapse, and overdose, the development of novel, alternative, or adjunct OUD treatment therapies is highly warranted. In this article, we review emerging evidence that suggests that cannabis may play a role in ameliorating the impact of OUD. Herein, we highlight knowledge gaps and discuss cannabis' potential to prevent opioid misuse (as an analgesic alternative), alleviate opioid withdrawal symptoms, and decrease the likelihood of relapse.

**Conclusion:** The compelling nature of these data and the relative safety profile of cannabis warrant further exploration of cannabis as an adjunct or alternative treatment for OUD.

## Introduction

The opioid epidemic has become an increasingly pressing problem with an estimated 26–36 million people abusing opioids around the world.^1^ At the time of this publication, the Centers for Disease Control reports that 115 people die every day of an opioid related cause in the United States, and more than 33,000 people lost their lives to an accidental opioid overdose in the United States in 2015 alone.^1–4^ The United States consumes 80% of the world's supply of prescription opioid analgesics (POAs), and opioid prescriptions have climbed by 300% since 1991.^5^ The rise in opioid prescriptions has also widened the demographic of individuals dying from opioid overdose; historically, overdose was most prevalent in urban, minority adolescent males; however, today these lethal effects are similar across race, gender, socioeconomic status, and geography.^7–11^ The spike in prescriptions has also directly contributed to an increase in the number of first-time consumers of illicit opioids (heroin, which is commonly laced with fentanyl or its analogs), which has continued to climb since the mid 1990's.^6^ Patients who become physically dependent upon POAs frequently switch to illicit opioids because POAs are more costly and/or difficult to obtain.^3,8,12,13^ However, ease of access is a dangerous tradeoff for the lethal risk that is associated with synthetic opioids. Fentanyl, for instance, is 100 times more potent than morphine, which partially explains why there was a 250% increase in synthetic opioid mortality between 2012 and 2015.^14,15^

This unprecedented public health crisis warrants the investigation of novel sustainable interventions which would directly address the current opioid misuse crisis, complement current treatment strategies, and prevent future misuse through alternative first line analgesics.

## Mechanistic Interactions between Cannabis and Opioids

The endocannabinoid and opioidergic systems are known to interact in many different ways, from the distribution of their receptors to cross-sensitization of their behavioral pharmacology. Cannabinoid-1 (CB1) receptors and mu opioid receptors (MORs) are distributed in many of the same areas in the brain, including but not limited to the periaqueductal gray,^16,17^ locus coeruleus,^18,19^ ventral tegmental area (VTA), nucleus accumbens, prefrontal cortex (PFC),^20^ central amygdala (CeA), bed nucleus of stria terminalis (BNST),^21^ caudate putamen (CP), substantia nigra, dorsal hippocampus, raphe nuclei, and medial basal hypothalamus.^22^ The extent of this overlapping expression and frequent colocalization of the CB1 and MOR provide clear morphological underpinnings for interactions between the opioid and cannabinoid systems in reward and withdrawal.^19,23^

There is a bidirectional relationship between MORs and CB1 receptors in the rewarding properties of drugs of misuse.^20,24–28^ That is, modulation of the CB1 receptor has profound effects on the rewarding properties of opioids, and *vice versa*. For example, MOR and CB1 receptors are reciprocally involved in the development of drug-induced conditioned place preference (CPP). Coadministration of a cannabinoid antagonist and morphine attenuates the development of morphine CPP,^26^ and coadministration of an opioid antagonist blocks tetrahydrocannabinol (THC)-induced CPP.^25^ Interestingly, microinjections of CB1 agonists into the medial PFC creates an aversion to doses of morphine that are normally rewarding (CPP), while CB1 antagonism in this brain region creates a rewarding effect of subthreshold morphine doses.^24^ In addition, administration of cannabinoids to MOR knockout (KO) mice produces a weaker CPP compared to wild-type animals,^22^ reviewed in Wills and Parker.^27^ This mutual involvement in reward is at least partially mediated by presynaptic cannabinoid and opioid disinhibition of dopamine neurons in the VTA, a well-characterized mechanism in the rewarding properties of drugs of misuse.^20^ Although these mechanisms have not been well studied in humans, one study has found CB1 upregulation in the reward pathway of individuals who use opioids, which supports a role for the endocannabinoid system in the development of opioid misuse.^29^

There is abundant support for the role of CB1 receptors in the rewarding effects of opioids and the amelioration of tolerance. However, the effects of endogenous and exogenous cannabinoids in opioid withdrawal are somewhat paradoxical: endogenous cannabinoids seem to have no role in somatic withdrawal,^27,30–32^ yet exogenous CB1 agonists readily alleviate somatic symptoms such as escape jumps, diarrhea, weight loss, and paw tremors.^28,33,34^ Endogenous cannabinoid tone within the amygdala is also involved in the affective component of opioid withdrawal, as blockade of CB1 receptors in the CeA or BNST ameliorates opioid withdrawal.^21^ The kappa opioid receptor (KOR) system may also play a role in cannabis' impact on the affective opioid withdrawal, given its pivotal contributions to dysphoria and negative effect.^35^ However, both KOR agonism (with U50, 488H^30^) and KOR antagonism (naloxone^31,32^) have both been shown to attenuate conditioned place aversion in CB1 KO mice.^30^ These contradicting data highlight the need for additional mechanistic insights into the involvement of the CB1 receptor in opioid reward and withdrawal.

## Cannabis as a First Line Analgesic

The primary use for both prescription opioids and cannabis is for analgesia. Currently, up to 90% of patients in state-level medical cannabis registries list chronic pain as their qualifying condition for the medical program.^36^ In an exhaustive review, the National Academies of Science and Medicine recently confirmed the efficacy of cannabis for chronic pain in adults.^36^ Interestingly, when given access to cannabis, individuals currently using opioids for chronic pain decrease their use of opioids by 40–60% and report that they prefer cannabis to opioids.^37–42^ Patients in these studies reported fewer side effects with cannabis than with their opioid medications (including a paradoxical improvement in cognitive function) and a better quality of life with cannabis use, compared to opioids. Despite the vast array of cannabis products and administration routes used by patients in states with medical cannabis laws, cannabis has been consistently shown to reduce the opioid dose needed to achieve desirable pain relief.^41,43^

One of the mechanisms that may explain the opioid sparing effects of cannabis is its ability to produce synergistic analgesia.^44–46^ In humans, subanalgesic doses of THC and morphine are equally unsuccessful at reducing the sensory or affective components of pain; however, when the same doses of THC and morphine are coadministered, they produce a significant reduction in the affective component of pain.^47^ These synergistic effects are also observed when patients using opioids for pain vaporize whole-plant cannabis, as opposed to experimentally administered isolated THC.^48^ Adjunct whole plant cannabis has no effect on the pharmacokinetics of opioids, which further supports a synergistic mechanism behind the opioid sparing and enhanced analgesia produced by cannabis.^48^ Furthermore, in pre-clinical models, coadministration of opioids and cannabinoids attenuates the development of opioid tolerance.^49,50^

Combined, these clinical and pre-clinical data suggest that analgesic synergy produced by coadministered cannabis and opioids could be harnessed to achieve clinically relevant pain relief at doses that would normally be subanalgesic. This strategy could have significant impacts on the opioid epidemic, given that it could entirely prevent two of the hallmarks of opioid misuse: dose escalation and physical dependence.

Because patients report substituting cannabis for several types of pharmaceutical drugs, including opioids, benzodiazepines, and antidepressants,^51^ analgesic synergy may not entirely explain the opioid-sparing effects of cannabis in pain patients. Economic and lifestyle considerations may also play a pivotal role in opioid sparing and substitution. Patients report that their reasons for substituting cannabis for other medications include less severe side effects, less withdrawal potential, ease of access, and better symptom management for their conditions.^52^

Although there is insufficient clinical literature to support the use of cannabis as a treatment for acute pain, there is a long-standing body of pre-clinical evidence that demonstrates the antinociceptive effects of cannabinoids in pain-free, drug-naive animals.^17,49,53–57^ The mechanisms of cannabinoid antinociception are remarkably similar to those of opioid analgesics. Both the CB1 and MOR are G-protein coupled receptors, and agonist-initiated disinhibition of GABA release in the descending pain pathway is just one example of overlapping antinociceptive mechanisms between these drugs.^17,23,58–62^ Evidence supporting the role of cannabis in acute, nonsevere pain management could lead to a substantial reduction in opioid prescription rates, thereby eliminating patient exposure to the risks of opioid dose escalation and physical dependence. This critical gap in the clinical literature and potential clinical impacts of this therapy warrants further exploration of the efficacy of cannabis for acute pain relief.

## Current Opioid Use Disorder Therapies and Their Shortcomings

The most prominent and pervasive problem in opioid use disorder (OUD) treatment is the prevention of drug relapse, which is extremely common during acute withdrawal (detoxification), as well as during protracted recovery after physical withdrawal symptoms have subsided.^63–66^ Abstinence-based protocols are particularly ineffective, as 85% of individuals relapse within 12 months of the initiation of treatment.^65^ In-patient residential treatment facilities do not appear to improve abstinence-based therapy, as relapse rates in this paradigm are as high as 80%, when measured 2 years after treatment initiation.^67^ Compared to abstinence, opioid replacement and medication-assisted therapies, which began in the 1960s, are more efficacious for relapse prevention; however, there are currently only four FDA-approved medications for the treatment of OUD.^68–71^ Off-label prescription medications such as benzodiazepines and antiemetics are also common, but these therapies are largely directed at symptom management during acute detoxification rather than relapse prevention.^72^ In this review, we focus on the most widely used OUD therapies, their shortcomings, and the bottlenecks to accessing them.^11^

Methadone, a full MOR agonist, was approved by the FDA in 1974 to aid in opioid cessation.^9,73^ Individuals enrolled in consistent dose methadone maintenance programs are more likely to stop using nonprescribed opioids than individuals not enrolled in the maintenance program.^74^ Although methadone has an encouraging safety profile,^75^ it carries some risk for misuse and mortality when the dose exceeds the patient's level of tolerance.^76,77^ Withdrawal symptoms from methadone mimic those of other opioids when stopped abruptly or tapered too quickly, and these symptoms last up to 3 weeks longer than withdrawal from other opioids.^9,78,79^ There are only 1590 methadone distributers in the United States, which are highly regulated clinics that are concentrated in urban areas, creating geographical disparities in OUD treatment.^10,11,79^ In addition to geographical barriers, these clinics frequently have stringent and stigmatizing compliance requirements, such as daily visits and frequent urine screenings for illicit drugs.^11,80^ Although these barriers to treatment could potentially be addressed through concerted efforts to expand access, 40% of patients still relapse within 1 year of initiating methadone therapy.^67^

Buprenorphine (Subutex) is a partial MOR agonist and KOR antagonist that can reduce withdrawal symptoms, cravings, and additional opioid use.^76,81,82^ The inclusion of naloxone in some buprenorphine formulations (Suboxone, Zubsolv) is intended to reduce misuse by precipitating withdrawal when it is used intravenously,^82,83^ and despite the presence of naloxone, there is still some risk for misuse and overdose.^77,83,84^ The inclusion of naloxone can also induce withdrawal when administered too soon after the most recent dose of other opioids.^63,67,85^

Unlike methadone, Suboxone offers a primary care approach to medication-assisted therapy, as it can be dispensed by a pharmacy rather than a specialized clinic.^86,87^ However, only 3% of physicians possess the additional Drug Enforcement Agency credentials required to prescribe buprenorphine,^76,88^ and there are strict limits on the number of patients they are permitted to serve.^89^ Buprenorphine-licensed physicians also tend to be concentrated in larger cities, leaving 46.8% of counties in the United States, especially rural areas and the Midwest, with a shortage in convenient access to these treatment options.^88,90,91^ While long-term treatment retention with buprenorphine or Suboxone is not as well characterized as methadone, a Swedish study has shown retention rates of up to 75% following a year of buprenorphine/Suboxone treatment.^92^ However, a 24-week clinical trial in the United States reveals that buprenorphine retention is only 46%.^93^

Evidence suggests that the most effective tool for relapse prevention is medication-assisted pharmacotherapy, combined with social support.^76,78,94,95^ Because of the overwhelming evidence that supports this concurrent treatment model, there is little rationale to deviate from this approach. However, expanded access to these therapies is highly warranted, as are novel and alternative therapies which improve efficacy, diminish geographical disparities, and eliminate the need for specialty physicians.^96^

## Cannabis During Acute Opioid Withdrawal

The first barrier to overcoming OUD is getting patients through the acute withdrawal period, or detoxification. Although pharmacotherapies such as methadone and buprenorphine are largely successful and widely utilized for this purpose, there are shortcomings to this approach, which are highlighted above.^9,76,78,80,82,83,87,89–91,97^ In May of 2018, the FDA approved the use of lofexidine, an alpha-2 adrenergic receptor agonist for acute (14 day) opioid withdrawal. Lofexidine provides substantially more symptom relief than placebo; however, the comparative efficacy of lofexidine in combination with long-acting opioid agonists or opioid antagonists is still being characterized.^71,98–100^

There is also nascent evidence that suggests that cannabis may be an efficacious tool during the acute opioid withdrawal period. Numerous pre-clinical studies have shown that cannabis and cannabinoids decrease opioid withdrawal symptoms.^6,33,34,97,101–103^ Although this evidence supports the use of cannabinoids as a possible treatment in OUD treatment,^28^ conflicting evidence demonstrates that CB1 agonism increases the rewarding properties of opioids^22,102^ and may actually increase the severity of opioid withdrawal symptoms.^18,104^ These conflicting data highlight the need for a mechanistic characterization of CB1 agonism as a therapeutic target for opioid withdrawal, a need that is further substantiated by the pharmacology of CB1 antagonism. For instance, some studies show that acute administration of SR-141716A, a CB1 antagonist, can reduce opioid withdrawal; however, this effect is profoundly affected by the experimental conditions.^22,105^ Because this effect can be recapitulated in CB1 KO mice, CB1 antagonism only partially mediates these effects.^102^ To complicate the story further, the administration of cannabidiol (CBD), a very promiscuous phytocannabinoid with at least a dozen mechanisms of action, also alleviates naloxone-precipitated withdrawal in morphine tolerant rats.^106–112^

Although the mechanisms by which cannabinoids alleviate opioid withdrawal are complex and unclear, some reports suggest that cannabis may alleviate opioid withdrawal in humans.^18,113^ For instance, patients engaging in medication-assisted detoxification from opioids reported using cannabis when opioid maintenance doses were not high enough to prevent withdrawal and cravings.^114^ However, some individuals reported that cannabis was often ineffective and sometimes worsened overall severity of the withdrawal symptoms. Because the phytochemical makeup and cannabinoid content of cannabis have a significant effect on subjective human experiences,^115^ it is plausible that these variable experiences are the result of variable phytochemistry in cannabis products that are self-selected by study participants. Unfortunately, blinded, placebo-controlled clinical trials evaluating the efficacy of cannabis, either alone or as an adjunct therapy for acute opioid withdrawal, are lacking. This is not entirely surprising, given cannabis' status as a Schedule I substance in the United States, which precludes federal funding to investigate cannabis as a medication-assisted therapy.

Unlike whole-plant cannabis, dronabinol, an FDA-approved analog of THC, has been evaluated for opioid withdrawal relief in a placebo-controlled study in patients receiving the opioid antagonist naltrexone. Low-dose adjunct dronabinol improved the tolerability of symptoms such as insomnia, reduced appetite, and reduced energy levels during opioid detoxification, whereas adverse events such as tachycardia were reported at higher dronabinol doses.^113,116,117^ In many studies, cannabinoids were safe and tolerable when coadministered with an opioid or opioid replacement medication.^47,113,118–120^ However, the comparative efficacy of dronabinol or other cannabinoids versus traditional replacement therapies such as methadone or buprenorphine remains to be elucidated. Given the efficacy and tolerability of Sativex (a whole-plant cannabis derivative) for pain and spasticity, investigation of adjunct Sativex for opioid withdrawal is warranted.^121–123^

Like opioids, chronic cannabis exposure induces the development of tolerance, physical dependence, and withdrawal symptoms during abstinence. Patients commonly report that cannabis withdrawal symptoms, most commonly anger, aggression, irritability, anxiety, decreased appetite, weight loss, restlessness, and sleeping difficulties,^124–129^ are similar to those produced by nicotine withdrawal.^129^ Comparatively, the magnitude and severity of cannabis withdrawal are significantly and substantially more benign than opioid withdrawal.^20,126^ In addition, and unlike opioids, cannabinoid withdrawal and subsequent relapse are nonlethal after periods of abstinence. The reduced intensity of cannabinoid withdrawal symptoms compared to opioids could at least partially be explained by the prolonged period of metabolization of cannabinoids in the body,^102^ contributing to the mounting support for cannabis as a harm-reduction tool to combat OUD.

## Cannabis as a Harm Reduction Tool in OUD

Pre-clinical evidence suggests that the CB1 receptor plays a critical role in opioid reward. Cannabinoid antagonism reduces the rewarding properties of opioids and prevents reinstatement of drug seeking.^105,130,131^ However, these effects were not reproducible in human clinical trials.^132–134^ Unlike CB1 antagonism, CB1 agonism may play a role in OUD treatment. Several studies have shown that adjunct cannabis decreases opioid consumption or prevents opioid dose escalation.^37–42,121,135^ Although these findings are promising, several other studies have shown that cannabis use either has no impact on opioid consumption or may increase nonmedical opioid use.^136–138^

The mechanisms underlying cannabis alteration of opioid consumption are yet to be determined; however, there is significant pre-clinical evidence which suggests that CBD, one of the most prevalent cannabinoid molecules in cannabis, plays a critical role. CBD does not have reinforcing effects in rodents, which supports its low potential for misuse.^16,139^ CBD has been shown to reduce the rewarding aspects of multiple drugs of abuse, such as cocaine, amphetamine,^16^ and nicotine.^140^ Administration of CBD also attenuates morphine CPP and cue-induced reinstatement of heroin self-administration in rats, without creating any aversive or rewarding effects on its own.^106,141–143^

These findings provide promising rationale for the use of CBD in opioid relapse prevention in humans. In fact, pilot clinical studies have shown that in individuals recently abstinent from heroin, CBD reduces heroin craving.^142^ This effect occurs as soon as 1 h after administration and lasts for up to 7 days. Adjunct CBD appears to be safe and tolerable, as 400 and 800 mg oral CBD administration does not intensify the effects of intravenous fentanyl or create any adverse effects.^118^ Because CBD is neither intoxicating nor rewarding and has an extremely large therapeutic window and impressive safety profile, the use of CBD to inhibit opioid craving has great therapeutic potential.

Adjunct cannabis use alongside current treatment strategies could help to improve the number of individuals engaging in OUD treatment, as well as increase treatment retention rates. Both dronabinol and intermittent whole-plant cannabis appear to increase the length of time patients remain in treatment for OUD.^6,113^ However, chronic cannabis consumption during naltrexone treatment was ineffective at improving treatment retention, highlighting the need for further research into the dose and frequency of cannabis use in OUD treatment retention and relapse prevention.^144^ Although the ubiquitous and ever-growing regulated cannabis markets across North America could potentially address the aforementioned shortcomings in OUD treatment accessibility and retention, there are currently very few addiction and recovery centers that have embraced concurrent social support and cannabis-assisted OUD treatment.^51^ This is unsurprising given the lack of empirical evidence to support this approach, and the lack of federal research funding that would support this work.

In addition to the clinical and experimental observations outlined above, epidemiological investigations in U.S. states with legal cannabis have provided insight into the promising role for cannabis in the opioid crisis. The implementation of both medical and adult-use cannabis laws appears to have a significant impact on opioid consumption and overdose. These states experience a 23% reduction in nonfatal opioid overdoses, as measured at hospital emergency departments.^145^ By analyzing death certificates, Bachhuber et al. found a 24% reduction in the annual rate of fatal opioid overdoses in the first year following medical cannabis legalization,^146^ an effect that gets larger the longer a state has had legal cannabis (33% in California, which has had medical use since 1996 and the lowest rate of opioid overdose fatalities in the country).^146,147^ This finding was also seen in data from the FARS, which demonstrates a similar drop in mortalities of opioid positive automobile accidents in states with implemented cannabis legalization for individuals aged 21–40.^148^ The mechanisms underlying cannabis' ability to reduce opioid hospitalization and mortality are unclear; however, analysis of the Medicare Part D prescription drug program has unveiled the possibility that cannabis may be serving as an analgesic alternative to opioids for individuals living in these states.^149^ The number of filled POAs is substantially lower in states with the most liberal cannabis laws, where there are 3.742 million fewer daily doses than in states with the most prohibitive laws.^150^

These epidemiological impacts are not exclusive to opioid prescriptions, hospitalizations, and mortality; the U.S. economy could also benefit from expanded cannabis legalization. Opioids cost patients and insurance companies upwards of 2.6 billion dollars in healthcare costs annually.^151^ While cannabis is still federally illegal, and in most cases dispensary purchases are not eligible to be covered under any healthcare insurance plan, states with legalized cannabis have seen significant decreases in Medicare Part D prescription drug spending, including, but not limited to, prescription opioids.^149,152–155^ Reductions in spending from Medicare Part D were over $165 million dollars.^149^ If cannabis were removed from Schedule I of the Controlled Substance Act and more patients had access to cannabis, savings from pharmaceutical costs incurred by the Medicare Part D prescription plan are projected to continue to climb.^149^

## Shortcomings of Cannabis in Medication-Assisted Therapy

Although the literature thoroughly supports the safety and tolerability of cannabis,^38,118,142,156^ there is conflicting evidence for its efficacy as a treatment for opioid misuse. Throughout the history of methadone administration, patients have reported that cannabis provides relief from opioid withdrawal symptoms, as well as breakthrough pain and anxiety.^119^ However, other evidence demonstrates that cannabis does not relieve withdrawal symptoms for individuals undergoing methadone tapering, and some participants even reported increased severity of their withdrawal symptoms.^104^ All the participants in the latter study procured their own cannabis and reported smoking as the route of administration. Because the dose of cannabinoids and phytochemical makeup of whole-plant cannabis have significant impacts on physiological responses (such as tachycardia) and subjective experiences (such as anxiety), additional research is needed to characterize maximally efficacious treatment protocols.^116,157^ When used to treat opioid withdrawal symptoms, undesirable side effects also occur in a dose-dependent manner for the FDA-approved cannabinoid dronabinol.^113^ The homogenous and consistent formulation of this pharmaceutical combined with the logistical ease of prescribing the drug may make it more feasible than whole-plant cannabis for clinical trials on cannabinoid alleviation of opioid withdrawal symptoms and relapse prevention.

Despite the promising results of reducing or maintaining a consistent opioid dose, it is plausible that the substitution of one rewarding substance (opioids) for another (THC) could be problematic, leading to cannabis use disorder (CUD). In 2016, ∼1.4–2.9% of adults over the age of 18 in the United States met criteria for CUD.^79^ With revisions to the criteria of substance use disorders in 2013, ∼19% of individuals who use cannabis throughout their lifetime would eventually meet the *Diagnostic and Statistical Manual of Mental Disorders*, Fifth Edition (DSM-5) criteria for CUD.^153^ The interpersonal or employment hardships experienced by these individuals that resulted in the meeting of DSM criteria may have simply been due to the legality of cannabis use; that is, a false CUD diagnosis is less likely to occur in the postprohibition era, when patients are no longer breaking the law.

Risks of CUD seem to be correlated with higher THC concentrations,^153^ which is a valid concern in legal markets where average THC potency is upward of 20%.^158^ Recreational users of cannabis have historically consumed cultivars higher in THC and lower in CBD, due to the desired intoxicating effects of THC.^38^ Medical users, however, have turned to cultivars higher in CBD and lower in THC in an attempt to optimize the medicinal benefits of cannabis.^38,153^ Although misuse potential is a valid concern, it is notable that the misuse liability of cannabis is very low.^159^ One possible approach to alleviate the concern of misuse is the concurrent administration of opioid antagonists. This approach seems to reduce the rewarding properties, but not the hyperphagia or withdrawal-relieving properties of THC.^160–164^ These data suggest that combined cannabis and opioid-antagonist therapy could be an effective tool against OUD, while also minimizing the risk for CUD. Because cannabis does not carry the risk of fatal overdose, the use of cannabis as a harm-reduction treatment in the opioid epidemic warrants further investigation.

## Summary and Future Directions

The opioid overdose epidemic is arguably the worst public health crisis in U.S. history. At the time of this publication, more people are dying than at the peak of the AIDS epidemic, and for the first time, drug overdoses outnumber automobile and handgun deaths.^165^ A continental crisis of this magnitude warrants the immediate implementation of novel strategies that prevent opioid misuse, overdose, and death.

Growing pre-clinical and clinical evidence appears to support the use of cannabis for these purposes. The evidence summarized in this article demonstrates the potential cannabis has to ease opioid withdrawal symptoms, reduce opioid consumption, ameliorate opioid cravings, prevent opioid relapse, improve OUD treatment retention, and reduce overdose deaths. Cannabis' greatest potential to positively impact the opioid epidemic may be due to its promising role as a first line analgesic in lieu of or in addition to opioids. The comparative efficacy of cannabis alone or in conjunction with current medication-assisted OUD therapies is not well characterized. However, no other intervention, policy, pharmacotherapy, or treatment paradigm has been as impactful as cannabis legislation has been on the rates of opioid consumption, overdose, and death.

Many of the barriers that prevent people from accessing traditional OUD treatment do not apply to cannabis therapy, and access to cannabis medicine is rapidly growing as more U.S. states roll back prohibition. However, a major barrier in universal patient access and improvement in the opioid epidemic is cannabis' status as a Schedule I controlled substance.^166^

Undoubtedly, more high-quality clinical evidence is needed to further support the use of cannabis to combat OUD; however, federal grant funding that would support these types of clinical trials is currently outside the scope of interest of the National Institutes of Health (because of Schedule I, cannabis is federally considered to have no medical benefit). Patients, healthcare providers, and regulating bodies would all greatly benefit from additional evidence that fills in massive gaps in the knowledge base about the utility of cannabis for OUD treatment: dosing, cannabinoid content and ratios, bioavailability, contraindications, misuse liability, route of administration, and many other questions remain. Even the clinical work that has been conducted thus far may have little validity in the modern landscape of legalized cannabis; all federally-funded cannabis research in the United States is conducted using a single source of cannabis (NIDA drug supply), which is notoriously low in potency and quality, and does not resemble the staggering phytochemical variability in whole-plant cannabis products in regulated state markets.^36^ These barriers to research funding and access to “real world” cannabis for clinical research directly contribute to our inability to address the opioid epidemic with what appears to be a safe and efficacious tool.

In light of the evidence presented in this article, and despite a lack of FDA approval, some U.S. states and private treatment centers have already begun to include cannabis as a part of OUD treatment protocols. The state of New Jersey recently added OUD to their list of qualifying conditions for participation in the state's medical cannabis program.^167,168^ Some private treatment centers are also citing the benefits of harm reduction, which greatly outweigh the risks of cannabis use during the first 28 days of recovery, a critical time period for patient survival.^76^

Many clinicians remain skeptical of cannabis as a viable treatment option, either due to the stigma surrounding cannabis use or the belief that there is not enough clinical evidence for them to feel confident providing patients with cannabis recommendations.^169^ This is unsurprising, given that 85% of recent medical school graduates still receive no education whatsoever about cannabis throughout their training, residencies, or fellowships.^170^ As the evidence in this field accumulates, it will be critically important to widen opportunities for clinicians to participate in Continuing Medical Education programs, which include the harm reduction and medical benefits that cannabis could provide. Evidence-based opioid prescription and cannabis recommendation practices are a critical component of continuing education, so that clinicians can continue to uphold their Hippocratic oaths to “do no harm.”

## Abbreviations Used

BNST: bed nucleus of stria terminalis
CB1: cannabinoid 1
CBD: cannabidiol
CeA: central amygdala
CPA: Conditioned Place Aversion
CPP: conditioned place preference
CUD: cannabis use disorder
DSM: *Diagnostic and Statistical Manual of Mental Disorders*
FARS: Fatality Analysis Reporting System
KO: knockout
KOR: kappa opioid receptor
MOR: mu opioid receptor
NIDA: National Institute of Drug Abuse
OUD: opioid use disorder
PFC: prefrontal cortex
POAs: prescription opioid analgesics
THC: tetrahydrocannabinol
VTA: ventral tegmental area
